# Safety climate in hospitals: A cross‐sectional study on the perspectives of nurses and midwives

**DOI:** 10.1111/jonm.13551

**Published:** 2022-03-02

**Authors:** Manela Glarcher, Karin Kaiser, Patrick Kutschar, Nadja Nestler

**Affiliations:** ^1^ Institute of Nursing Science and Practice Paracelsus Medical University Salzburg Austria

**Keywords:** hospital, midwives, nurses, patient safety, safety climate, safety culture

## Abstract

**Aims:**

To explore nurses' and midwives' perspectives of safety climate in Austrian hospitals as measurable elements of safety culture and to identify areas of quality improvement.

**Background:**

Due to close contact with patients, nurses and midwives play a vital role in ensuring patient safety.

**Method:**

An online survey among 713 nurses and midwives was conducted, using the 19‐item Safety Climate Survey (SCS). To answer the survey, a 5‐point Likert scale was provided with higher ratings indicating a more positive safety climate.

**Results:**

Results demonstrate a positive safety culture (MD 4.09, SD 0.53). Significant group differences in overall safety climate score could be found regarding nurses and midwives in managerial positions, between gender and participants age with low effect size. High item missing rates focus aspects on management/leadership, institutional concerns, leadership by physicians, and handling of adverse events. In addition, these items present the lowest ratings in safety climate.

**Conclusion:**

Results indicate potentials for optimization in the areas of leadership communication and feedback, the handling of safety concerns, and visibility or improvement of patient safety strategies.

**Implications for Nursing Management:**

A regular, standardized safety climate measurement can be a valuable tool for nurse managers and (political) decision‐makers to manage patient safety initiatives.

## 1 BACKGROUND

Health care facilities are complex organisations with a high potential in medical errors, which can be influenced by the existing organisational culture (Ettl & Offenberger, 2014). Safety culture can be delineated as a general aspect of the organisational culture and is described as a phenomenon that includes norms, values, and beliefs of the organisation, not of the individual. Safety culture is reflected in the way safety and risks are dealt with and in the behaviour of those involved (Ausserhofer et al., 2012). The term ‘safety culture’ is understood to mean a shared set of knowledge, values, and symbols that increases the capacity of the organisation to improve patient safety (Schrappe, 2017). In order to assess the safety culture, the safety climate is used as a measurable parameter (Gehring et al., 2015). Safety climate as a critical element in terms of patient safety and quality care describes a psychological phenomenon, defined as employees' perception of safety‐related aspects in their working environment at a certain point of time (Seibert et al., 2020). It influences staff behaviour, which in turn affects patient outcomes, and therefore can be expected to impact patient safety. Research findings demonstrate a reduction in medication errors (Fan et al., 2016), as well as lower 30‐day readmission rates for patients treated in units where the safety climate is rated high by health care staff (Mascherek & Schwappach, 2017). A strong safety climate is characterized by two‐way safety communication, management support, and appreciation for safety and safety systems and is considered a predictor of nurses' safety performance (Manapragada et al., 2019). An essential basic prerequisite for high‐quality and safe care is appropriate staffing in nursing (McHugh et al., 2021). With the aim to ensure the development of a safety climate among employees, three basic characteristics have been identified: formation of a safe working environment by senior management (1), development of a common understanding of safety in the work environment, which includes adequate staffing (2), and effective dissemination of safety information (3) (Lin et al., 2017).

In order to identify, prospectively control, and monitor changes in safety‐relevant aspects, safety climate measurements have been used in recent years. As a quantitative form of assessment, it provides a snapshot of safety culture manifestation (Manser, Brösterhaus, & Hammer, 2016) and can generally be considered an early indicator that offers information about possible risks before an accident has occurred (Pfeiffer & Manser, 2010). Assessment of safety climate can be performed differently using international instruments in health care organisations, that is, the ‘Hospital Survey on Patient Safety Culture’ (HSoPS) (Sorra & Nieva, 2004), quantifying safety climate at the level of the organisation, the ‘Safety Attitudes Questionnaire’ (Sexton et al., 2006), and the ‘Safety Climate Survey’ (SCS) (Sexton & Thomas, 2003), both focussing on the level of a health care team or unit (Manser, Frings, et al., 2016), with the SCS being the shortest and thus taking the least time for participants to response (Sexton & Thomas, 2003).

Historically, the publication ‘To Err is Human: Building a Safer Health System’, published by the Institute of Medicine (IOM) in 1999, to which at that time around 44,000 to 98,000 people die annually from preventable adverse events (IOM, 2001), gained international importance, and marked the starting point in safety initiatives in health care organisations (Alsalem et al., 2018). The World Health Organization (WHO) considers patient safety to be the reduction of the risk of unnecessary harm to health to an acceptable minimum (WHO, 2011). In Austria, in year 2013, the Patient Safety Strategy 2.0, an Austria‐wide framework, pursues the goal of creating awareness for safe care and reducing risks in the care and/or treatment process among all actors (decision‐makers, health care professions, and population). Corresponding goals and measures to ensure patient safety in health care organisations were derived in policy development (1), organisational development (2), personnel development (3), and public development (4) (Federal Ministry Republic of Austria, 2018). Based on current national strategy as well as previous research, an initiative to strengthen the safety culture in an Austrian hospital company was started. The purpose of this study was to explore registered nurses' (RNs) and midwives' perspectives of the safety climate in hospitals and to identify areas of quality improvement.

As previous studies have shown that the assessment of safety climate is significantly influenced by the gender and age of the participants (Jiang et al., 2019) as well as by a leadership position and the hospital unit (Gehring et al., 2015), we included these characteristics in the rational of our study. We hypothesized that there would be group differences in nurses and midwives reported safety climate rating based on sociodemographic characteristics, age (a), gender (b), hospital unit (c), professional experience (d), and/or managerial position (d). Hence, the following research questions were pursued:

1. How do registered nurses and midwives describe safety climate in their hospital and are there any differences by select sample characteristics?
2. What areas of quality improvement can be identified from the survey results in the sample and what factors are associated with safety climate?

## 2 METHODS

### 2.1 Design and sample

A cross‐sectional, descriptive, exploratory, online survey was performed using the internationally recognized Safety Climate Survey (SCS) and the online tool Lime Survey. Population frame consisted of 3704 nurses and midwives in one hospital organisation in Austria. A convenience sample of registered nurses (RNs) with a diploma in nursing or a bachelor degree and midwives was applied, irrespective of professional experience, level of employment (full‐time/part‐time position), and managerial position (yes/no) from all clinical disciplines (e.g., surgery, internal medicine, and gynaecology) who had direct patient interaction. Nurses or midwives who were exclusively involved in organisational and administrative tasks and had no direct patient contact were excluded.

### 2.2 Data collection

In cooperation with the hospital management, information was provided to the participants via the company's internal magazine and their homepage as well as in meetings with hospital managers. All eligible 3704 employees received an email invitation to voluntarily participate in the survey, which was generated anonymously in the survey tool Lime Survey. After written informed consent, participation in the online survey was possible. In terms of questions or uncertainties, the research team could be contacted via email or phone.

Prior to data collection, a written pre‐test (*n* = 34 participants from the target group) was conducted to check the comprehensibility of the survey and included answer options, as well as the feasibility of an online survey in the company. Based on the feedback to participants' written comments, the email cover letter, the collection of sociodemographic data, and organisation's spam filters were adjusted.

### 2.3 Instrument

The German translation of the original Safety Climate Survey (SCS) was used (Gehring et al., 2015). The total number of items in the German SCS are 19 of 5‐point Likert scale ranging from 1 = *strongly disagree* to 5 = *strongly agree* and an answer option ‘I can't say’. The survey is suitable for interviewing all health care staff in hospitals (Sexton & Thomas, 2003). Higher values in the participants' assessment correspond to a more positive safety climate. Psychometrics of the German survey correspond approximately to those of the original survey (Cronbach's α Original = 0.85, Kho et al., 2005, Cronbach's α German = 0.86) (Gehring et al., 2015). Due to the shortness of the survey, participants need about 10 min to answer all items. This aspect is a great advantage compared with other instruments, especially when interviewing health care staff (Stiftung Patientensicherheit, 2014).

To record participant's sociodemographic characteristics, age (years), gender, hospital unit (medical, surgical, or operating/recovery room), professional experience (categories in years), managerial position (yes/no), and a free field for further comments were added. Not all sociodemographic data were mandatory fields. Only the hospital unit had to be specified.

### 2.4 Statistical analysis

The survey data from the online tool Lime Survey were exported into IBM SPSS Statistics for Windows, version 27, and descriptive statistics, frequencies, and percentages were calculated. Negatively pooled items were recoded to ensure that higher item values represented a more positive safety climate. We calculated measures to fully describe differences and variations in the safety climate, namely, the scale mean index, that is, the sum of all response values divided by the number of items, item means, standard deviations (SD), and the percentage of problematic responses (PPR) (Singer et al., 2008). PPR refers to the percentage of respondents, who scored ≤2 on the 5‐point Likert scale. Accordingly, a low PPR is an indicator of a high safety climate (Singer et al., 2009). A reported PPR of more than 10% in one item is interpreted as an indication of a minor patient safety climate (Mascherek & Schwappach, 2017). To determine group differences and an influence of participants sociodemographic characteristics on the overall SCS scale mean index, *t* tests for independent samples as well as one‐way analysis of variance (ANOVA) were carried out. Homogeneity of the error variances was assessed by Levene's test, Cohen's *d* was used to calculate and interpret effect sizes for all significant findings, and Tukey post hoc test in ANOVA was used to examine differences among sample means for significance; 95% confidence intervals (CI) were calculated for differences in means. Statistical tests were two‐tailed with a significance level of *p* < 0.05.

## 3 RESULTS

### 3.1 Sample characteristics

In summary, 713 nurses and midwives participated in the study (response rate 19.2%) from whom the majority were female (80.4%). The mean age of the participants was 41.5 years (SD 10.24). Around 15% of the survey participants also had a managerial position. The majority of nurses and midwives had more than 20 years of professional experience (44.3%), 13.8% 15 to 20 years, 13.1% 10 to 15 years, 13.5% 5 to 10 years, and 15.2% up to 5 years of professional experience. In summary, 427 participants were working on medical hospital units (59.9%), 216 (30.3%) on surgical units, and 70 (9.8%) were employed in operating areas or recovery rooms.

### 3.2 Nurses' and midwives' perspectives of safety climate

Nurses' and midwives' overall perspective of safety climate was positive. The mean value of SCS scale index was 4.09 (SD = 0.53) and varied for individual items between 3.44 and 4.64. The highest mean values were given in nurses' and midwives' own responsibility for patient safety, in item 17 ‘The personnel in this clinical area take responsibility for patient safety’ (4.64, SD = 0.58) and item 9 ‘I know the proper channels to direct questions regarding patient safety’ (4.55, SD = 0.72). The lowest ratings focused safety concerns in item 7 ‘Management/leadership does not knowingly compromise safety concerns for productivity’ (3.44, SD = 1.42) and item 16 ‘I believe that most adverse events occur as a result of multiple system failures, and are not attributable to one individual's actions’ (3.51, SD = 1.13). Items related to participants' learning from mistakes and dealing with errors, personnel briefing activities, their own responsibility, and knowledge for patient safety as well as management consideration of suggestions made by health care staff were perceived positively (4.05 to 4.64) by nurses and midwives. Items that included aspects of listening, communication, and receiving feedback from leaders, as well as addressing staff implementation/adherence to patient safety measures, achieved item ratings of 3.44 to 3.97.

All items demonstrated missing responses (0.80 to 23.80%). In four items (items 7, 14a, 15, and 16), there were more than 100 missing values. These focused management/leadership and institutional concerns as well as leadership by physicians and adverse events. In addition, these items showed the lowest ratings in safety climate. The PPR of the overall scale was 14.85%. In summary, 13 out of 19 items showed a PPR higher than 10% and varied from 3.30% in item 17 to 32.90% in item 7 (Table 1).

**TABLE 1:** Participants' perspectives on safety climate

Items in SCS	Mean	SD	PPR (%)	Missing (%)
1. The culture of this clinical area makes it easy to learn from the mistakes of others.	4.05	0.95	10.50	5.3
2. Medical errors are handled appropriately in this clinical area.	4.09	1.01	12.30	5.5
3. The senior leaders in my hospital listen to me and care about my concerns.	3.55	1.14	26.30	6.2
4. The physician and nurse leaders in my areas listen to me and care about my concerns.	3.97	1.02	14.90	1.1
5. Leadership is driving us to be a safety‐centred institution.	4.00	1.08	12.60	7.6
6. My suggestions about safety would be acted upon if I expressed them to management.	4.33	0.94	8.30	7.3
7. Management/leadership does not knowingly compromise safety concerns for productivity.	3.44	1.42	32.90	21.9
8. I am encouraged by my colleagues to report any safety concerns I may have.	4.23	0.94	7.80	4.2
9. I know the proper channels to direct questions regarding patient safety.	4.55	0.72	3.60	1.4
10. I receive appropriate feedback about my performance.	3.80	1.17	19.40	1.7
11. I would feel safe being treated here as a patient.	4.09	0.93	9.20	5.3
12. Briefing personnel before the start of a shift (i.e., to plan for possible contingencies) is an important part of safety.	4.56	0.79	4.80	5.6
13. Briefings are common here.	4.12	1.11	13.80	8.3
14. I am satisfied with the availability of clinical leadership (please respond to all three):				
Physician	3.81	1.10	19.40	14.2
Nursing	4.39	0.91	7.90	4.3
Pharmacy	4.10	1.00	10.80	1.7
15. This institution is doing more for patient safety now, than it did one year ago.	3.85	1.11	15.30	23.8
16. I believe that most adverse events occur as a result of multiple system failures, and are not attributable to one individual's actions.	3.51	1.13	25.30	19.4
17. The personnel in this clinical area take responsibility for patient safety.	4.64	0.58	3.30	1.0
18. Personnel frequently disregard rules or guidelines that are established for this clinical area.	3.93	1.15	17.90	4.5
19. Patient safety is constantly reinforced as the priority in this clinical area.	4.43	0.85	5.80	0.8
SCS scale mean index (19 items)	**4.09**	**0.53**	**14.85**	**7.19**

### 3.3 Group differences in nurses' and midwives' safety climate perspectives

Calculation of group differences demonstrated more positive safety climate ratings in nurses and midwives holding a managerial position (*T* = 2.818, *p* = 0.005), in females (*T* = 3.245, *p* < 0.001), and between age categories (*F* = 3.488, *p* = 0.016). Nurses and midwives in the age of 50 years and older scored significantly more positive than those under the age of 30 years (*T* = 3.126, *p* = 0.016). Details for all group differences are presented in Table 2.

**TABLE 2:** Group differences in SCS mean values

Variable	Mean (*SD*)	*n*	*p* value	Test statistics (*df*)	CI 95%_Diff._	*d*_Cohen_
Managerial position						
Yes	4.22 (0.48)	110	*p* = 0.005	*T* = 2.818 (690)	[0.05; 0.26]	0.293
No	4.07 (0.54)	582				
	Missing response	21				
Gender						
Male	3.92 (0.61)	106	*p* < 0.001	*T* = 3.245 (133.43)	[0.08; 0.33]	0.388
Female	4.13 (0.51)	573				
	Missing response	34				
Professional experience in years (categories)						
<5	4.10 (0.48)	107	*p* = 0.737	*F* = 0.499 (4)	n.s.	n.s.
5 < 10	4.03 (0.61)	95				
10 < 15	4.10 (0.50)	92				
15 < 20	4.08 (0.57)	97				
>20	4.11 (0.53)	311				
	Missing response	11				
Hospital unit						
Medical	4.10 (0.52)	427	*p* = 0.860	*F* = 0.151 (2)	n.s.	n.s.
Surgical	4.07 (0.56)	216				
Operating/Recovery room	4.10 (0.58)	70				
	Missing response	0				
Age in years (categories)						
≤30	4.02 (0.51)	141	*p* = 0.016	*F* = 3.488 (3)	n.a.	0.247^a^
31 ≤ 40	4.05 (0.54)	183				
41 ≤ 50	4.09 (0.56)	203				
>50	4.20 (0.51)	166				
	Missing response	20				
Post hoc test for significant group differences in age categories (≤30 vs. >50)						
≤30	4.02 (0.51)	141	*p* = 0.016	*T* = 3.126 (305)^b^	[0.03; 0.34]	0.358
>50	4.20 (0.51)	166				

Abbreviations: CI 95%, confidence interval 95%; *d*_Cohen_, effect size by Cohen's *d*; Diff., difference of means; *df*, degrees of freedom; *F*, statistic ANOVA; n.a., not applicable; n.s., not significant; SD, standard deviation; *T*, statistic *T* test for independent samples.

^a^ Based on eta^2^ = 0.015.

^b^ Based on pairwise *T* test for independent samples.

## 4 DISCUSSION

This is the first known companywide study in Austrian hospitals using the Safety Climate Survey (Sexton & Thomas, 2003) in nurses and midwives with the aim to explore safety climate, assess group differences, and identify areas of patient safety improvement. Despite our efforts to inform the participants about the survey via team meetings, newsletters, and email response rate in our study was low at 19.2% with high missing responses, an overall mean value of 4.09 (SD = 0.53), and items mean vary from 3.44 to 4.64. PPR‐analysis showed that 13 out of 19 items are higher than 10%, with three items (items 3, 7, and 16) above 20%. Highest ratings were shown in item 17 (4.64), lowest ratings in item 7 (3.44). Comparable data demonstrated in a Swiss study (Gehring et al., 2015), in which 3153 health professionals, including 1321 nurses, completed the survey. Within a response rate of 64%, the nurses in this sample reported mean SCS of 3.75, which was slightly lower than in the Austrian sample of nurses and midwives (4.09). Scores at item level in the Swiss study ranged from 3.18 to 4.38. In line with our findings, the highest item rating was observed in item 17 (4.18). Also, present studies' results of PPR proved similar to Gehring et al. (2015). The Swiss PPR of 11.76% was slightly lower than in our study (14.85%). At item level, 14 out of 19 items showed a PPR higher than 10%, and two items (also items 7 and 16) returned a PPR higher than 20%.

However, our Austrian sample focused only on perspectives of the safety climate of nurses and midwives and therefore cannot be fully likened to the results of the Swiss survey. Furthermore, it should be noted that the sample size shows an influence on the level of PPR and the influence of each individual is stronger in small groups (Mascherek & Schwappach, 2017). This aspect could have an impact on our sample of 713 participants, and the results are therefore only comparable with the Swiss sample to a limited extent.

The SCS was also used in a 2‐year national quality improvement programme in Switzerland. Scale means from health care professionals' perspective (physicians = 1075, nurses = 2089, others = 599) were measured at two times, before (3.80, SD = 0.50) and after (3.90, SD = 0.60) surgical checklist implementation with significant improvement but low effect size (Mascherek et al., 2016).

In Turkey, nurses' perception of safety climate (*n* = 350) was quite low with a safety climate scale mean of 3.50 (SD = 0.62), compared with the Austrian and Swiss sample. In addition, 15 out of 19 items reached a PPR higher than 10% with the lowest item mean in item 3 (2.64, SD = 1.18), the same as in our study, followed by item 10 (3.22, SD = 1.18). The highest ranking was given in item 12 (4.14, SD = 0.91) (Dirik & Seren Intepeler, 2017).

Considering mean scores at item level in our study, the participants pointed out that their hospital as well as leadership is safety‐centred, the organisational culture enables learning from mistakes of others, briefings are common, and medical errors are handled in an appropriate manner. But on the other side, they give lower safety climate ratings to aspects of listening, communication, and receiving feedback from leaders than in aspects of their own responsibility. These results are consistent with insights from another study, where nurses reported that management did not address staff concerns, so these assumptions that patient safety is not a priority for management (Wagner et al., 2019).

As demonstrated in a hospital safety climate study including the perspective of nurses in four European countries, nurses perceived patient safety, and their ability to report incident data is correlated with dimensions of ‘organisational learning’ and ‘feedback and communication about error’ (Gurková et al., 2020). Areas of quality improvement in this Pan‐European survey can be identified in ensuring safe productivity by leadership as well as in their communication within team members and giving feedback to nurses and midwives, physician accessibility to staff, and transparency of/or improved patient safety strategies. Comparable studies also show potential for optimization in the area of organisation learning/continuous improvement with regarding hospital safety culture (Mascherek et al., 2016; Saleh et al., 2015). Ensuring effective communication, feedback, committed leadership, and an environment that focuses on learning from errors emerge as appropriate ways to do this (Okuyama et al., 2018).

According to the findings of previous studies (Jiang et al., 2019), our results show small significant differences in safety climate ratings between gender and ages. In particular, participants with an age above 50 years evaluate the safety climate significantly more positively than participants who were younger than 30 years. It is therefore surprising that professional experience is not an influencing characteristic in the assessment of the safety climate in this study. In addition, higher safety climate ratings have been demonstrated in nurses and midwives hold on managerial positions. Their perception of safety climate was more positive than those of participants without this position. Direct patient contact and seniority are mostly associated with a more critical perception of safety‐related aspects. In particular, working directly with patients generally makes patient safety issues more prominent over time. Study results illustrate that maintaining a safety climate by providing feedback on errors and maintaining open communication is positively related to the frequency of incident reporting. Nurses perceive higher levels of patient safety when there is enough staff on the ward (Saleh et al., 2015). Otherwise, nurses in larger teams perceive a lower safety climate. This correlation can possibly be explained by a decrease in the frequency of communication in larger groups and an associated loss of communication. However, it is also possible that nurses in larger teams consciously engage less (Seibert et al., 2020).

A higher culture of patient safety is associated with better patient outcomes (Fan et al., 2016; Mascherek & Schwappach, 2017). High‐quality care in hospitals aim to provide multidisciplinary care to patients with minimal risks (Okuyama et al., 2018). Organisational culture and the active support throughout hospital managers play a key role in promoting and maintaining safety culture (Levine et al., 2020). Above all, a connection between organisational culture and safety climate is shown in the view of failure as system errors and not as individual errors, which is why organisational processes should be continuously analysed and improved. Nurses and midwives hold on managerial positions must be strengthened in anticipating the weak points in the organisational system and in forcing organisational team learning. Patient safety workshops to promote a culture of safety, as well as team training to raise managers' personal awareness and challenge routines show themselves to be suitable ways of doing so (Kanerva et al., 2013).

### 4.1 Strengths and limitations

Data collection was conducted online during working time, using an internationally approved and practicable tool. Nevertheless, we had a certainly high number of missing answers in our survey. This could either be an indication that the questions themselves were not well understood by the participants and reflect a misunderstanding, or that ‘risky items’ were deliberately not answered due to a fear of consequences. Also, the aspect of social desirability response, which is often noticeable in surveys, that is, that participants may give biased answers that are coherent with prevailing social values (Polit, 2021), cannot be precluded.

Currently, the theoretical basis of measurement tools to assess safety climate has been limited and demonstrates potential for optimization. In addition, further research is needed to evaluate the relationship between safety culture and patient outcomes (Alsalem et al., 2018). However, the safety climate as measurable element of safety culture is not a static phenomenon (Seibert et al., 2020), but is subject to constant organisational change. The results of our survey therefore refer exclusively to the time of data collection and may already have changed over time. Another note of care should be taken when interpreting and generalizing the data to other hospitals as data analysis and results are limited to nurses and midwives in one hospital operator in Austria. Furthermore, due to recommendations of the works council in the hospital, it was not possible to evaluate the data between these two professions separately. Nevertheless, we have no reason to assume that our sample differs essentially from the general population in terms of gender, age, or professional experience. Participation in the survey was voluntary, which can result in the involvement of particularly motivated health care staff and thus represent a possible misrepresentation of the results.

## 5 CONCLUSIONS

This is one of the very rare studies in German‐speaking countries to analyse safety culture and nurses and midwives' perspectives of safety climate. While safety culture was evaluated rather positive overall, several essential improvable explicit aspects of safety climate were identified. Nurses and midwives rated their own responsibility for patient safety higher than items related to safety concerns, whereas the lowest ratings refer to listening, communication, and received feedback from staff in a managerial position. Although stating only weak effects, results indicate that safety climate perspectives might vary by gender, age, and managerial position of participating nurses and midwives, while neither professional experience, nor hospital unit proved significant. Results might further demonstrate, that, despite existing national demands, efforts to record safety culture as an indicator of patient safety and quality of care are not yet sufficiently recognized.

## 6 IMPLICATIONS FOR NURSING MANAGEMENT

Management can significantly influence nurses' safety behaviours by engaging leaders as role models for safety, promote an open, two‐way channel for safety‐related communication, and acknowledge and support safety systems in the workplace (Manapragada et al., 2019). Through a coordinated interaction of leadership management, environmental factors and work processes, the fundamentals for learning from errors among nurses and midwives can be established and an improvement in patient safety supported. Training of nurses and midwives to deal with errors and to improve their communication should be an integral part of continuing educational activities in hospitals. Leadership has an important task to enable health care staff in building a culture of safety (Fischer et al., 2018; Levine et al., 2020; WHO, 2011). As the largest professional group within hospitals nurses' and midwives' perspectives of safety climate can be considered as a leading indicator of the currently dominant safety culture. Regular, standardized safety climate measurement may offer valuable information to hospital leaders and (policy) decision‐makers to maintain as well as continuously improve the culture of patient safety.

## CONFLICT OF INTEREST

The authors declare no conflict of interest.

## AUTHOR CONTRIBUTIONS

MG was responsible for the study design and data collection. MG, KK, and PK were responsible for the data analysis. MG, KK, PK, and NN were responsible for the manuscript writing.

## ETHICS STATEMENT

Participation in the survey was voluntary and anonymous. It is not possible to track back answers in the survey to individual persons. The right to withdraw from the survey at any time was pointed out and consent to the general data protection regulations was obtained. Furthermore, the worker's council of the hospital organisation was informed and gave their agreement. Permission to use the original SCS was obtained from Patient Safety Switzerland.

## Data Availability

Data are openly available in a public repository that does not issue DOIs.
